# Deep Learning-Based Fine-Tuning Approach of Coarse Registration for Ear–Nose–Throat (ENT) Surgical Navigation Systems

**DOI:** 10.3390/bioengineering11090941

**Published:** 2024-09-20

**Authors:** Dongjun Lee, Ahnryul Choi, Joung Hwan Mun

**Affiliations:** 1 Department of Biomechatronic Engineering, College of Biotechnology and Bioengineering, Sungkyunkwan University, Suwon 16419, Republic of Korea; mouse87@skku.edu; 2 Department of Biomedical Engineering, College of Medicine, Chungbuk National Univeristy, Cheongju 28644, Republic of Korea

**Keywords:** surgical navigation system, deep learning, surface registration, coarse registration, registration error

## Abstract

Accurate registration between medical images and patient anatomy is crucial for surgical navigation systems in minimally invasive surgeries. This study introduces a novel deep learning-based refinement step to enhance the accuracy of surface registration without disrupting established workflows. The proposed method integrates a machine learning model between conventional coarse registration and ICP fine registration. A deep-learning model was trained using simulated anatomical landmarks with introduced localization errors. The model architecture features global feature-based learning, an iterative prediction structure, and independent processing of rotational and translational components. Validation with silicon-masked head phantoms and CT imaging compared the proposed method to both conventional registration and a recent deep-learning approach. The results demonstrated significant improvements in target registration error (TRE) across different facial regions and depths. The average TRE for the proposed method (1.58 ± 0.52 mm) was significantly lower than that of the conventional (2.37 ± 1.14 mm) and previous deep-learning (2.29 ± 0.95 mm) approaches (*p* < 0.01). The method showed a consistent performance across various facial regions and enhanced registration accuracy for deeper areas. This advancement could significantly enhance precision and safety in minimally invasive surgical procedures.

## 1. Introduction

In the field of biomedical technology, recent advances in surgical techniques and the development of sophisticated devices have considerably improved clinical outcomes [1,2]. Minimally invasive surgery, which offers benefits such as reduced physical stress, lower blood loss, shorter recovery periods, and decreased medical costs through smaller incisions, is gaining popularity [3,4]. Surgical navigation systems, which are indispensable in these procedures, provide surgeons with a precise internal view by displaying the three-dimensional placement of surgical tools in real time, based on preoperative medical images [5,6]. The accuracy of these systems is ensured through a registration process that precisely matches the 3D medical image space with the patient’s anatomy [7,8].

Two primary registration techniques are employed in surgical navigation systems: point registration and surface registration, each having unique characteristics and specific applications for aligning preoperative imaging with the patient’s anatomy during surgery. The point registration technique involves inserting fiducial markers beneath the patient’s anatomy [9]. This method calculates a transformation matrix by precisely locating the markers within both diagnostic imaging and the patient’s anatomy, enabling the correlation of these two regions. Although relatively accurate, this technique presents several disadvantages [10,11]. It necessitates additional preoperative imaging, which increases the patient’s exposure to radiation [10], and requires the implantation of fiducial markers in the patient’s anatomy until the completion of surgery, introducing infection risks at implantation sites [11].

In contrast, surface registration techniques estimate counterparts in medical images from patient surface contours for alignment, thus eliminating the need for fiducial markers [10,11]. This method is more efficient and particularly appropriate for minimally invasive surgery. The direct approach, adaptability, and suitability of surface registration for minimally invasive procedures have spurred research aimed at enhancing this technique [8,12,13,14]. Typically, surface registration is a two-step process involving coarse and fine registration. Coarse registration manages large rotational and translational variations between datasets, establishing a foundation for subsequent refinement. It typically relies on easily identifiable anatomical landmarks to achieve an approximate alignment. Fine registration further refines this initial alignment between preoperative and intraoperative points. This process is usually performed using the iterative closest point (ICP) algorithm, which iteratively minimizes discrepancies between point sets to enhance the alignment [15]. However, the ICP method faces challenges in identifying corresponding registration points across datasets without explicit point-to-point correspondence [12]. This issue largely results from the ICP’s heavy reliance on the quality of initial registration, where poor initial alignment can lead to substantial registration errors, as ICP tends to converge to local optima [16].

The necessity for accurate initial alignment has driven focused research into alternative methods beyond traditional anatomical landmark-based coarse registration. These initiatives typically concentrate on capturing a sufficient number of surface points to enhance the detail and accuracy of patient-image registration. Fan et al. [12,13] demonstrated the potential of 3D scanning technologies for coarse registration in surgical navigation. Their method uses a structured light 3D scanner to capture significantly more surface points than traditional landmark-based approaches, resulting in improved registration accuracy. This improvement was demonstrated by experiments that showed a reduction in target registration error (TRE)—the distance error between target points in the medical image and patient space after registration for points not used in the registration process. However, this scanner-based method introduces several critical limitations, including a complex calibration process due to the addition of the scanner’s coordinate system, which leads to residual rotational errors that exacerbate misalignment. Additionally, the large number of outliers among the collected points can increase registration errors [14,17]. Addressing the limitations of scanner-based methods, Li et al. [14] proposed an incremental registration method using an optical probe. This technique aims to acquire a more comprehensive point cloud of the patient’s facial region, employing constraints that ensure minimum distances between collected points and guarantee a certain percentage of facial surface coverage for uniform point acquisition. Although the method demonstrated significant improvements in registration performance, it relies heavily on the precision of point collection and constraint parameter settings, which can impact registration accuracy. Furthermore, the technique’s dependence on the operator’s skill in system operation and point collection introduces potential variability in results across different users. These studies underscore the ongoing challenges in achieving both accurate and efficient surface registration processes.

Recent advancements in machine learning, particularly in processing complex three-dimensional data, suggest potential avenues for enhancing both accuracy and efficiency. Deep-learning frameworks such as PointNet, DGCNN, and PointCNN have demonstrated their ability to handle point cloud data directly, eliminating the need for projection into higher-dimensional structures [18,19,20]. These techniques have been successfully implemented in fields such as autonomous driving, geospatial analysis, and virtual reality, proving their effectiveness in managing complex 3D data. Although deep learning has achieved success in many fields, its application in surgical navigation presents unique challenges [21,22]. One of the primary obstacles is the scarcity of large, labeled surgical datasets, which impedes the development of robust and generalized models. Moreover, real-time performance is crucial for intraoperative use, adding complexity to model design and implementation, as many current methods struggle to meet the computational demands required for real-time use in a clinical setting. Additionally, there remains a gap between synthetic training data and real-world application, as models trained on simulated data may not always perform optimally in actual surgical environments. Recent efforts by Yoo and Sim [23] have explored the potential of deep-learning techniques to enhance the accuracy of surgical navigation registration. Their method utilizes virtual 3D patient point clouds to train a deep-learning model in estimating relative 3D rotations and translations, enabling automated coarse registration without the need for identifying anatomical landmarks. However, their direct method of estimating spatial relationships between medical images and patient spaces struggles to obtain training data that cover possible positions and orientations. Additionally, their evaluation is confined to the coarse registration step, focusing solely on the surface registration error (SRE)—the distance error between patient points and their corresponding paired points in the medical image used during the registration process. For surgical procedures demanding sub-millimeter precision, these limitations necessitate further improvements.

The objective of our research is to propose a novel surface registration protocol that improves the accuracy of conventional surface registration in surgical navigation systems. Our approach integrates a deep learning-based refinement step between traditional coarse registration and ICP fine registration, thereby enhancing the existing pipeline within the established workflow. The innovative aspects of the proposed protocol include an automatic training set generation process that creates a comprehensive dataset with diverse simulated scenarios. Additionally, the protocol is designed to be trained preoperatively, encompassing a wide range of anatomical variations. It seamlessly integrates into existing surgical workflows and operates in real time during procedures. Through this approach, the proposed protocol bridges the gap between theoretical advancements in deep learning and practical applications in surgical navigation registration. The specific goals of the study are as follows:

1. Develop a novel surface registration protocol that integrates deep-learning methodologies with conventional model registration techniques to enhance accuracy in surgical navigation systems while maintaining existing clinical workflows.
2. Design a specialized deep-learning model architecture capable of processing sparse point clouds and predicting precise refinements for optimized registration.
3. Validate the proposed method within existing surgical navigation workflows through phantom-based studies, assessing both SRE and TRE to demonstrate improved accuracy.

## 2. Materials and Methods

### 2.1. Conventional Surface Registration Process

**Figure 1:**
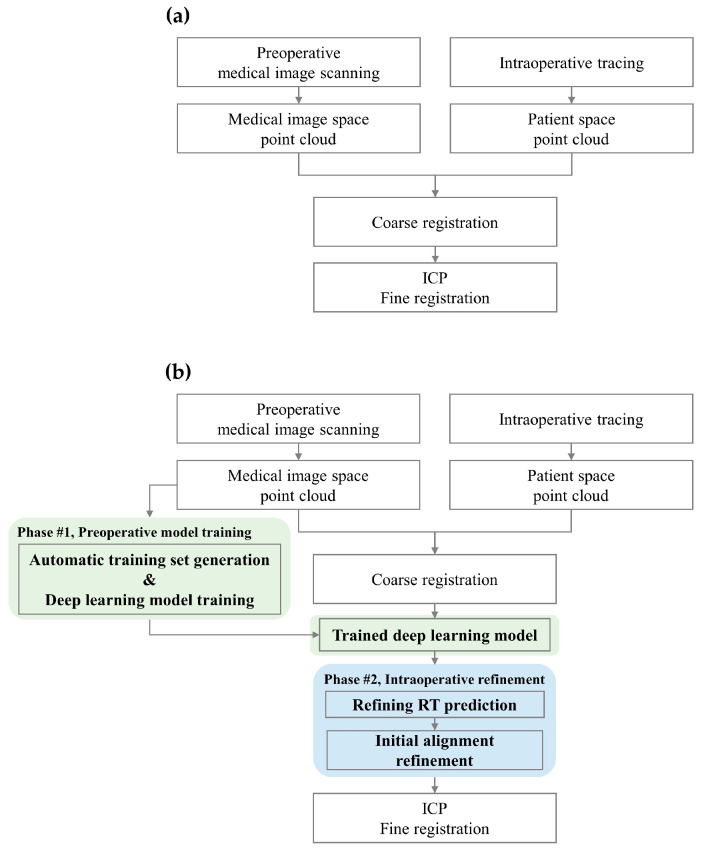
(**a**) Conventional surface registration process and (**b**) proposed surface registration protocol. In (**b**), the two phases of the proposed method are color-coded: green represents Phase #1: Preoperative model training, which includes automatic training set generation and deep learning model training, and blue represents Phase #2: Intraoperative refinement.

In surgical navigation systems, registration aligns point clouds derived from the preoperative medical image space and the intraoperative patient space. The conventional surface registration process is illustrated in Figure 1a.

#### 2.1.1. Medical Image and Patient Space Point Cloud Acquisition

Preoperative medical image scanning serves as the baseline for surgical navigation. Computed tomography (CT) scans produce high-resolution images of the target area, which are stored in the DICOM (Digital Imaging and Communications in Medicine) format. The marching cubes algorithm [24] is then used for three-dimensional surface reconstruction, extracting iso-surfaces from volumetric data. Subsequently, the vertices of the reconstructed 3D surface are extracted to create the medical image point cloud. Intraoperatively, the patient space point cloud is acquired using surgical navigation systems. For this purpose, an optical or electromagnetic tracking device equipped with a probe is utilized. During the surgical procedure, this probe collects spatial data points by capturing the patient’s anatomical features, representing both facial geometry and specific anatomical landmarks. These data points collectively constitute the patient space point cloud, which represents the actual anatomy of the patient in the operating room.

#### 2.1.2. Coarse Registration

**Figure 2:**
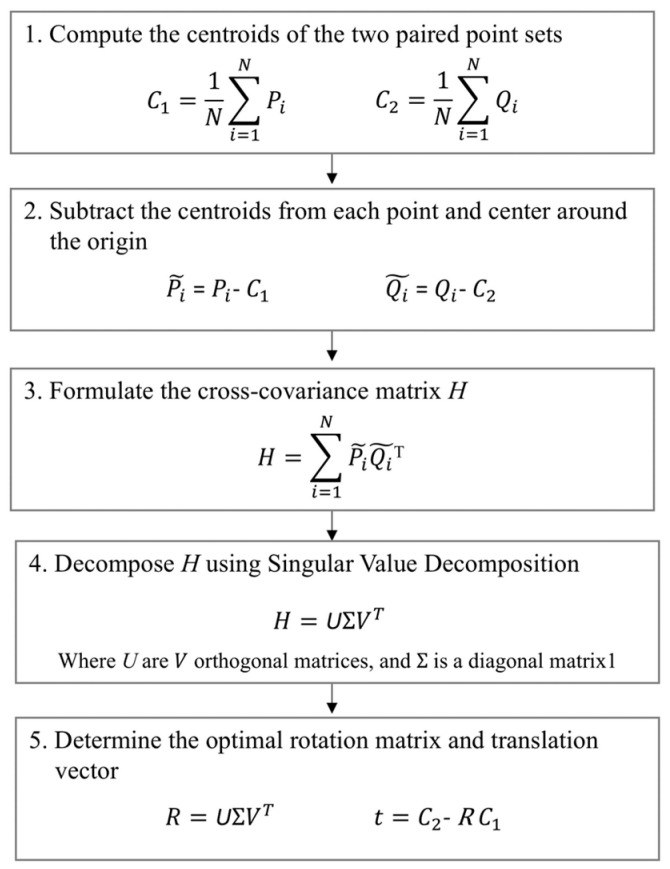
SVD-based paired point registration method.

The initial step in the surface registration process employs paired anatomical landmarks to align medical images. The standard singular value decomposition (SVD)-based paired point registration method [25] is used to perform paired point registration. This method aims to identify the optimal rigid transformation that minimizes the mean square error between matching points across two landmark sets (Figure 2). The process starts with the calculation of the centroids for both point sets. Each point in each set is then translated by subtracting its respective centroid, thus aligning the point sets to a common origin. This translation streamlines the calculation of the optimal rotation. The cross-covariance matrix (H) between the translated point sets is then computed, capturing the linear relationship between the two sets of points. Following this, the matrix is decomposed into three matrices (U, Σ, V) using SVD, which determines the principal directions of variation in the data. The optimal rotation matrix is determined using the results from SVD. This matrix aligns the two point cloud sets. The translation vector is then calculated by comparing the centroids of the target point set and the rotated source point set. The resulting transformation matrix is used to approximately align the points with those in the target. When implementing SVD-based coarse registration, it is crucial to consider the potential for localization errors in identifying anatomical landmarks, as inaccuracies can lead to significant deviations in the transformation matrix and result in suboptimal alignment [26,27].

#### 2.1.3. ICP Fine Registration

Following the coarse registration, fine registration is employed to enhance the alignment further. ICP iteratively refines the transformation between two point clouds by minimizing the distance between corresponding points.

It begins by identifying the closest point in the target point cloud for each point in the source point cloud. These pairs of points are regarded as temporary correspondences. Using these correspondences, the algorithm estimates a transformation (comprising both rotation and translation components) that optimally aligns the points. This transformation is computed using the SVD-based paired point registration method outlined in the coarse registration section. A key distinction in ICP is that this process is applied iteratively with determined point pairs, as opposed to using a fixed set of manually selected landmarks. Once calculated, this transformation is applied to the source point cloud, bringing it into closer alignment with the target. The algorithm continues until convergence is reached, typically defined as the point where the change in error between iterations falls below a predefined threshold, or when a maximum number of iterations is reached.

### 2.2. Proposed Surface Registration Protocol

In this study, we propose a novel surface registration protocol aimed at reducing the localization error of anatomical landmarks and enhancing the reliability of the initial coarse registration. The protocol integrates deep-learning techniques with conventional registration methods to address the misalignment between medical images and patient space and to predict the refinement matrix. The protocol consists of two main phases: (1) Automatic training set generation and deep-learning model training; (2) Refining the coarse registration using the trained model. This process is illustrated in Figure 1b.

**Figure 3:**
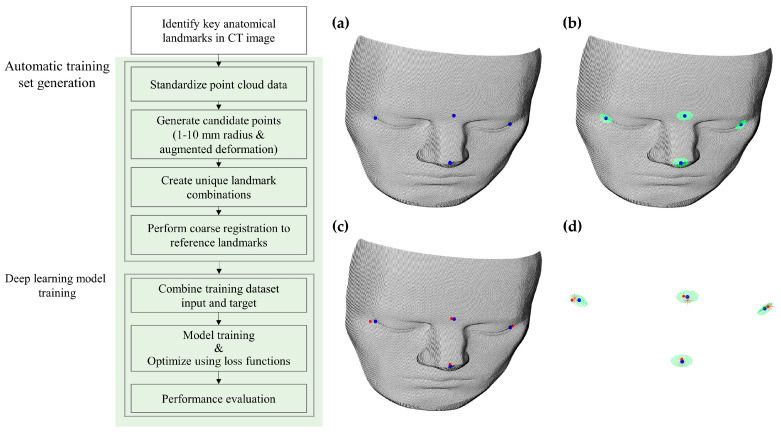
Automatic training set generation and deep-learning model training process. (**a**) Key anatomical landmarks in blue dots; (**b**) Candidate regions in green dots; (**c**) Simulated landmarks in red dots; (**d**) Coarse registration result between blue and red dots in red star-shaped points.

The initial phase involves constructing a comprehensive dataset specifically designed to train a deep-learning model. The dataset is automatically constructed with a large number of simulated anatomical landmarks introduced with localization errors, ensuring that it covers a wide range of scenarios likely to occur during surgery. The goal is to address the entire spectrum of potential misalignments and inaccuracies that could arise in actual surgical settings. Once the dataset is complete, a deep-learning model is trained to predict the necessary refinements to the initial coarse registration (Figure 3).

The second phase of the protocol involves applying a trained deep-learning model to enhance the coarse registration achieved through the SVD-based paired point registration method. The model predicts the transformation matrix required for precise alignment of the coarsely registered anatomical landmarks with their true paired points in the medical image. By refining the alignment prior to the fine registration step, this protocol aims to enhance the overall precision of the surgical navigation system.

#### 2.2.1. Automatic Training Set Generation

The training set generation process consists of creating a comprehensive dataset with thousands of unique combinations of simulated anatomical landmarks. The deep-learning model leverages this training set as a crucial component to comprehend the relationship between the coarse-registered geometry and the actual target. The ultimate goal of the model is to predict the transformation matrix that accurately aligns the coarsely registered anatomical landmarks with their precise counterparts on medical images.

- Standardization of point cloud data

The initial stage of generating training data involves applying principal component analysis (PCA) to 3D-reconstructed medical image point cloud data. Multiplying the PCA component onto the medical image allows for alignment of the eigenvector axes of the point cloud with a Cartesian coordinate system, ensuring a standardized orientation across datasets. Additionally, a scaling operation was carried out to position the medical image point cloud within a unit sphere, thereby normalizing the data. This step improved the model’s learning efficiency, accelerated convergence, and enhanced training stability.

- Identification of anatomical landmarks and candidate points generation

Key anatomical landmarks for coarse registration, such as the nasion, the tip of the nose, and the corners of the canthus, are identified from the medical image point cloud [28,29]. A range of 1 to 5 mm is known for potential identification inaccuracies during the landmark localization on a patient’s face using a navigation system [30,31]. Candidate regions are defined to illustrate the range of possible landmark positions due to inherent manual identification errors. To simulate more realistic and challenging situations [10,28], a localization error range expansion to 10 mm and an augmentation of the simulated tissue deformations were added [32].

- Generation of unique landmark combinations and coarse registration

A large-scale simulation process generates numerous unique combinations of anatomical landmarks, each consisting of corresponding point clouds that mimic the selection of landmarks within specified candidate areas. Imbalanced classes and biased datasets are recognized concerns in deep-learning model training, as they can lead to inaccurate results [33]. To generate a balanced and unbiased dataset, hierarchical sampling was utilized to ensure balanced representation across different anatomical landmark candidates, and duplicates were removed to eliminate redundant landmark combinations. For each simulation, the landmark points undergo coarse registration to paired reference anatomical landmarks using SVD-based methods to yield a transformation matrix. This matrix aligns the simulated landmarks with the reference landmarks as accurately as possible despite the presence of localization errors. The training dataset for the deep-learning model is constructed by combining the coordinates of the coarse-registered landmarks with their associated transformation matrices. Each entry in the dataset includes the coordinates of these landmarks and the transpose of the calculated transformation matrix, which serves as the ground truth for model training. This dataset covers a broad range of scenarios typical in surgical settings, encompassing various anatomical variations and potential errors in landmark identification. By integrating diverse data, the dataset ensures the model generalizes effectively to real-world applications.

#### 2.2.2. Deep-Learning Model

- Problem statement

Given two point clouds X = {x_i_ ∈ R^3^|i = 1, …, N} and Y = {y_j_ ∈ R^3^|j = 1, …, M}, where X represents the source point cloud of coarse-registered simulated patient anatomical landmarks and Y represents the target point cloud from candidate regions of localization error. The transformation T that best aligns X to correspondences on Y can be represented as T = (R, t), where R ∈ SO(3) is a 3D rotation matrix and t ∈ R^3^ is a 3D translation vector. We parameterize the rotation R using a unit quaternion q = [q_w_, q_x_, q_y_, q_z_]^T^ ∈ R^4^, of ||q|| = 1. The problem can be formulated as finding the optimal transformation T* that minimizes the alignment error between the transformed patient point cloud and its corresponding target medical points (Equation (1)).

(1) $$\left(R^{*},t^{*}\right)=\underset{\left(R,\;t\right)\;}{\mathrm{argmin}}\underset{x_{i}\;\in \;X}{\sum }D\left(Rx_{i}+t,\;\mathrm{mY}\left(x_{i}\right)\right)$$

Here, D is a Euclidean distance metric and mY(x_i_) is the corresponding point in Y for x_i_. The deep-learning model aims to predict this optimal transformation T* through an iterative refinement process.

- Encoder architecture

**Figure 4:**
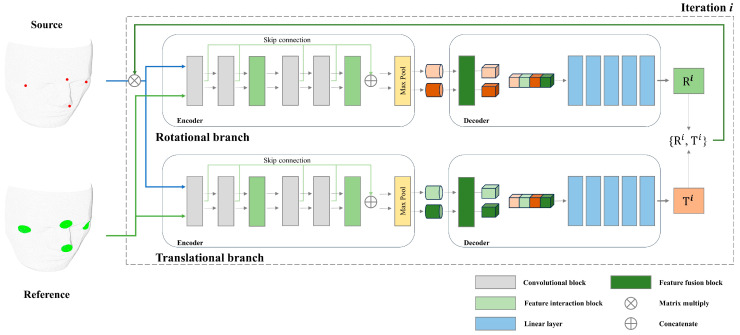
Deep-learning model architecture for coarse registration refinement.

The encoder uses a dual-branch design to separately handle rotational and translational components of the source and reference point clouds, as shown in Figure 4. This design preserves the distinct spatial representations characteristic of quaternion (rotational) and Euclidean (translational) spaces.

Each branch comprises a feature extractor that utilizes multiple 1D convolutional layers within a structured multi-layer perceptron (MLP) framework (3, 64, 128, 256, 512). These layers extract discrete point-wise features from both rotational and translational components of the anatomical landmarks. Each convolutional layer is succeeded by standardized regularizations including batch normalization and ReLU activation, which stabilize learning and introduce non-linearity [34]. To capture multi-level information, point-wise features undergo channel-wise max-pooling subsequent to each convolutional layer. The architecture integrates a point-wise feature interaction (PFI) module every two convolutional stages to enhance the exchange of low-level information between the source and reference point clouds. Skip connections are utilized to propagate low-level information and address the vanishing gradient problem. The encoder design is pivotal in extracting features from both source and reference point clouds, thereby setting the stage for precise alignment during the decoding and regression stages.

- Decoder architecture

The decoder processes the multi-level features extracted by the bi-branch encoder to produce accurate pose estimation for alignment. As depicted in Figure 4, it features two main components: the fusion module and the regression module. The fusion module merges feature vectors from corresponding hierarchical levels of both the rotational and translational branches of the encoder and processes them through three layers with hidden dimensions of (2048, 2048, 1024) to refine the interactions between the rotational and translational components while maintaining their distinct spatial characteristics. The resulting rotational and translational feature maps are then concatenated to form a unified representation that combines information from both branches. The concatenated features are then inputted into fully connected layers, preparing them for the final regression step.

The regression module estimates rotation and translation parameters for alignment. The network includes four blocks of linear layers with dimensions of 2048, 1024, 512, and 256, featuring regularizations. For rotation, the final layer outputs a normalized four-dimensional quaternion to represent valid rotation. The translation layer’s final output is a three-dimensional vector that represents the relative position between the centroids of the source and reference point clouds.

- Iterative refinement process

A key aspect of the utilized deep-learning model is its iterative refinement process, specifically engineered to incrementally enhance the alignment between the source and reference point clouds. The process begins with the model predicting an initial transformation, which includes both rotation and translation components, derived from the input point clouds. It then proceeds through a preset number of iterations. At each iteration, the predicted transformation updates the source point cloud. This transformed source point cloud, coupled with the original reference point cloud, provides the basis for the next iteration. The ultimate transformation, calculated by merging the transformations from all iterations as per Equation (2), reflects the cumulative transformation T, where T(i) represents the transformation at each iteration i, and n stands for the total number of iterations. This methodical approach enables the model to progressively sharpen its predictions, yielding more accurate alignments than those produced by single-step methods [35].

(2) T = T(n)× T(n −1)×⋯× T(1)

- Loss functions

The training of the deep-learning model involves multiple loss functions to ensure accurate registration of the point clouds. The total loss is computed as a weighted sum of these individual losses. Mean squared error (MSE) loss, or L2 norm loss, is applied to both the rotational and translational components [36]. For rotational accuracy, the predicted quaternions are converted to Euler angles prior to loss calculation. The MSE loss assesses the discrepancy between the predicted and the actual values, promoting precise alignment in rotation and translation. Chamfer distance loss is utilized to quantify the alignment error between the transformed source (predicted) and the target point clouds, enabling the model to achieve precise geometric alignments [37].

During training, the total loss is accumulated across all iterations, and gradients are calculated for backpropagation. The optimizer then updates the model parameters based on these gradients to minimize the total loss and enhance registration accuracy.

(3) $$L2\;norm\;loss=\frac{1}{n}\overset{n}{\underset{i=1}{\sum }}{\left(y_{i}-f\left(x_{i}\right)\right)}^{2}$$

(4) $$CD\left(S_{1},\;S_{2}\right)=\frac{1}{\left|S_{1}\right|}\underset{\left\{x\;\in S_{1}\right\}}{\sum }\underset{y\;\in {\;S}_{2}}{\min}{\left|\left|x\;-\;y\right|\right|}_{2}^{2}+\frac{1}{\left|S_{2}\right|}\underset{\left\{y\;\in S_{2}\right\}}{\sum }\underset{x\;\in {\;S}_{1}}{\min}{\left|\left|x\;-\;y\right|\right|}_{2}^{2}\;$$

- Hyperparameters and optimizer

The Adam optimizer is employed to effectively manage large-scale data and sparse gradients. The optimizer’s hyperparameters are set with an initial learning rate of 10^−3^ and a weight decay of 10^−4^ [38]. The model’s layers and nodes are meticulously fine-tuned through extensive testing to optimize performance and prevent overfitting [39]. The networks are trained using Pytorch for 500 epochs, in batches of 50, on a single NVIDIA RTX 3080 GPU. A StepLR scheduler modifies the learning rate every 100 epochs with a decay factor of 0.1, systematically reducing the learning rate to enhance the training process [40].

#### 2.2.3. Refining RT Prediction and Initial Alignment Refinement

As shown in Figure 1b, the trained deep-learning model is applied intraoperatively between the coarse registration and ICP fine registration steps. This process provides refined initial alignment for ICP fine registration while seamlessly integrating the process into existing surgical workflows.

- Landmark types and coordinate systems
  1. Patient’s anatomical landmarks: These landmarks, identified in the patient space, incorporate localization errors.
  2. Reference anatomical landmarks: These are the ground truth points serving as a common reference for alignment, assumed to be precise in the medical image coordinate system.

During a surgical procedure, patient anatomical landmarks are identified in the patient space and registered to the reference anatomical landmarks using SVD-based coarse registration. This initial registration yields a transformation matrix T_PAT_, which approximately aligns the landmarks but is not perfect due to localization errors in real-world trials. The pre-trained deep-learning model then predicts a refinement transformation T_REF_ based on the input of coarse-registered patient landmarks. This step corresponds to the “Refining RT prediction” in Figure 1b. The predicted T_REF_ serves to adjust the coarse-registered patient landmarks to align more accurately with their counterparts in the CT image. The final transformation that aligns the patient’s surface with the CT image is defined as T_REF_ × T_PAT_. This combined transformation provides a more accurate initial alignment for the subsequent ICP fine registration step, as shown in the “Initial alignment refinement” stage of Figure 1b.

### 2.3. Phantom-Based Validation Study

A validation study was conducted to assess the performance of the proposed surface registration protocol for registering internal structures compared to the conventional method. Four head phantoms (two females and two males) covered with silicone masks replicating human skin hardness (shore hardness 25 and 30 A) were manufactured for the study [41]. Target frames mimicking lesions were inserted into the phantoms. These frames were fabricated using a 3D printer (ZPrinter 650, 3D Systems, Rock Hill, SC, USA) and designed as a square grid with three columns and five rows, featuring a 35 mm interval between center lines. Ten target points were marked at 10 mm intervals in each row. Two-dimensional (2D) tomographic images of these soft phantoms were obtained using a Siemens Somatom Definition Edge CT scanner (Siemens Healthcare, Forchheim, Germany), with optimized scanner settings to achieve high-resolution images featuring a pixel size of 0.5527 mm in a matrix of 512 × 512 and a slice thickness of 0.55 mm.

The deep-learning model was trained using data generated from the four phantoms. For each phantom, 5000 simulated landmark picking trials were conducted on the 3D reconstructed CT image. The trials were divided into training, validation, and testing sets in an 8:1:1 ratio, resulting in 16,000 training samples, 2000 validation samples, and 2000 testing samples across all four phantoms. To comprehensively assess the proposed protocol, point cloud data from patients were acquired using an optical tracking system. This system included four optical cameras (Optitrack Flex 13, Natural Points, Corvallis, OR, USA) and a passive surgical probe (Northern Digital Inc., Waterloo, ON, Canada), which is typical of the setups commonly utilized for point cloud acquisition [42]. To evaluate the protocol’s effectiveness across different facial regions, five unique acquisition patterns were established. These were based on anatomical regions frequently employed in craniofacial surface registration [43,44]: Pattern #1 (Forehead + nose), Pattern #2 (Entire face), Pattern #3 (Face borderline), Pattern #4 (Forehead), and Pattern #5 (Above lip, Forehead + nose + around orbits). The design of the study entailed five independent trials for each pattern, leading to a total of 25 trials per phantom. With four phantoms used, the study comprised a total of 100 trials.

**Figure 5:**
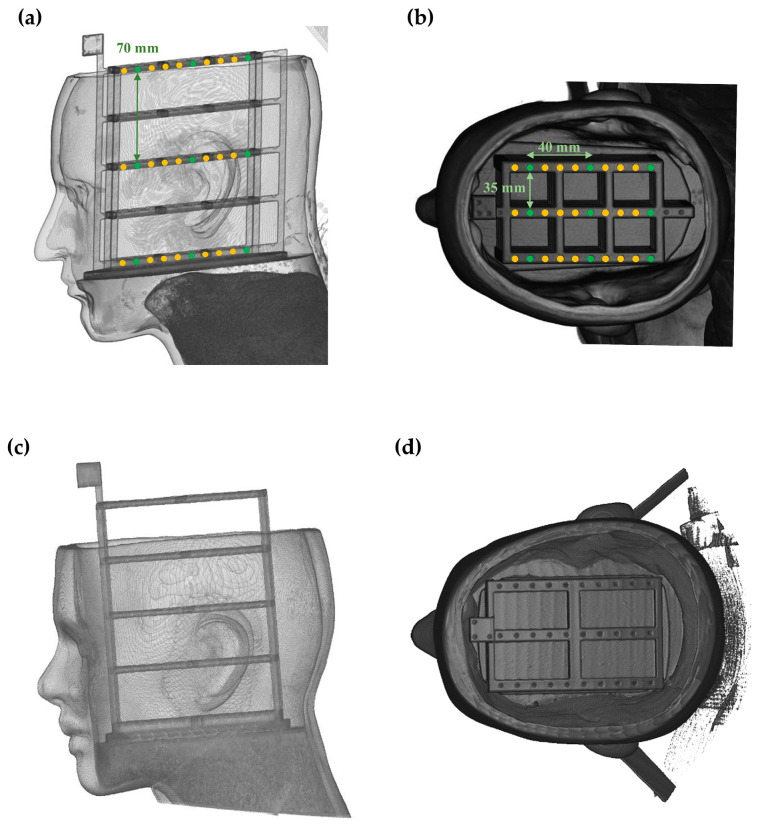
CT image of soft head phantom with silicone mask and internal target frame. (**a**) Male Soft phantom in sagittal view; (**b**) Male Soft phantom in transverse view; (**c**) Female Soft phantom in sagittal view; (**d**) Female Soft phantom in transverse view.

As depicted in Figure 5, a total of 27 internal target regions were deployed to assess registration errors on targets for each trial. These regions were uniformly distributed across the top (1st column), middle (3rd column), and bottom (5th column) layers, with nine targets per layer. Each layer’s nine targets consisted of three targets positioned at 30 mm, 70 mm, and 110 mm from the surface, arranged in three rows. The 27 target positions were analyzed statistically, aggregating data from all phantoms, point cloud patterns, and trials. This resulted in a total of 2700 data points for TRE analysis (27 targets × 4 phantoms × 5 patterns × 5 trials). The performance of proposed protocol was evaluated by comparing the TRE and SRE across the three models (Model 1, Model 2, and the proposed model). The TRE and SRE were calculated using the following equations:

(5) $$TRE=\left|Target_{medical}P-Target_{patient}P\right|$$

(6) $$SRE=\frac{\sum_{i=1}^{n}\left|Medical_{P_{i}}-Patient_{P_{i}}\right|}{n}$$

Statistical analysis was conducted to test the null hypothesis that there are no significant differences in TRE and SRE among Model 1 (conventional method [15]), Model 2 (Yoo and Sim [23]), and the proposed model. A one-way analysis of variance (ANOVA) was employed to examine the differences between models, with a significance level set at *p* < 0.05. In cases where ANOVA indicated significant differences, post-hoc comparisons were performed using Tukey’s honest significant difference (HSD) test [45]. Tukey’s HSD test was chosen for its ability to control Type I error rates when making multiple pairwise comparisons. For Tukey’s HSD test, statistical significance levels were set at *p* < 0.05 and *p* < 0.01. The analysis examined the overall performance (2700 target points for TRE; 21,504 surface points for SRE), performance across five different acquisition patterns (540 target points per pattern), and performance by distance from the surface at 30 mm, 70 mm, and 110 mm (900 target points per distance). All statistical analyses were performed using PASW Statistics version 18 (SPSS Inc., Chicago, IL, USA).

## 3. Results

**Figure 6:**
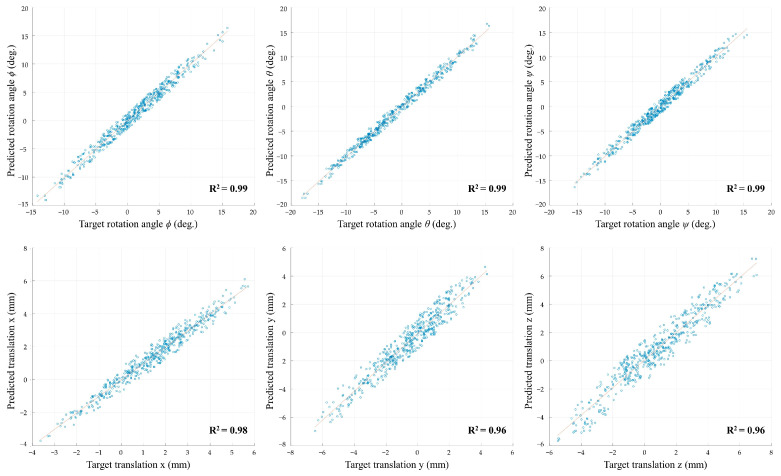
Scatter plots showing the correlation between the target and predicted rotation angles (φ, θ, ψ) and translation values (x, y, z) with R^2^ values.

Figure 6 demonstrates the correlation between the target and predicted rotation angles (φ, θ, ψ) and translation values (x, y, z). For the rotation angles, R^2^ values of 0.99 for all three angles (φ, θ, ψ) indicate a very strong correlation between the target and predicted rotations. Similarly, the translation values exhibited R^2^ values of 0.98, 0.96, and 0.96 for the x, y, and z axes, respectively, with an average R^2^ value above 0.98 across all six plots.

Table 1 presents the root mean square error (RMSE) values for the predicted rotation angles (Rx, Ry, Rz) and translation values (Tx, Ty, Tz) across five different dataset folds. The average RMSE values for the rotational components are 1.037° (SD = 0.054°) for Rx, 0.807° (SD = 0.048°) for Ry, and 1.094° (SD = 0.031°) for Rz, demonstrating the model’s ability to predict rotational angles with average errors below 2 degrees. For the translational components, the model achieves average RMSE values of 0.479 mm (SD = 0.123 mm) for Tx, 0.698 mm (SD = 0.048 mm) for Ty, and 0.706 mm (SD = 0.052 mm) for Tz, all below 1 mm.

**Figure 7:**
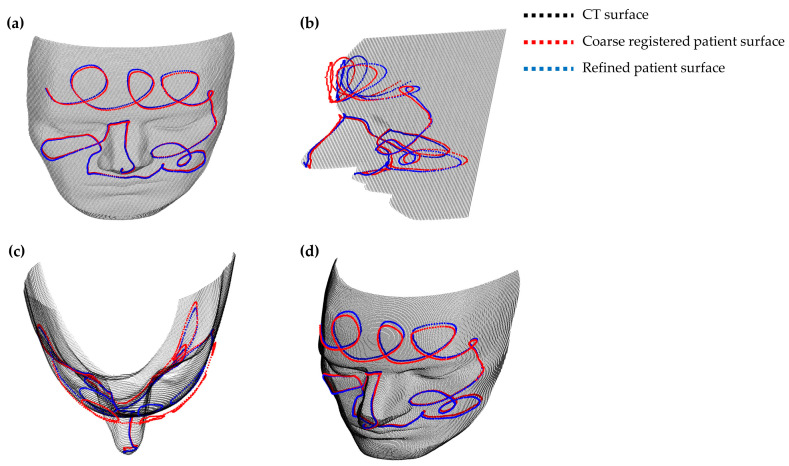
Visual comparison of conventional coarse registration (red) and the proposed deep learning-based refinement (blue) against the CT surface (gray). (**a**) Frontal view; (**b**) Sagittal view; (**c**) Transverse view; (**d**) Isometric view.

Figure 7 illustrates the results of a representative coarse registration trial, comparing the SVD-based coarse registration method (red) with the proposed deep learning-based refinement (blue). The CT surface, shown in gray, serves as the reference. Four distinct views are provided to assess the alignment comprehensively. Notable discrepancies are observed in the conventional coarse registration (red), especially in the nasal and periorbital regions, critical for accurate registration. In contrast, the refined patient surface (blue), utilizing the deep-learning model’s predicted transformation matrix, shows significantly improved alignment with the CT surface.

To evaluate the registration performance, a comparison was made between the proposed protocol, the conventional method, and the model proposed by Yoo and Sim [23]. The SRE values after ICP fine registration for these models (Table 2) were analyzed in terms of the minimum, maximum, average, and standard deviation. The conventional approach (Model 1) showed an average SRE of 0.534 mm (SD = 0.025 mm), ranging from 0.482 mm to 0.596 mm. The method by Yoo and Sim [23] (Model 2) demonstrated an average SRE of 0.544 mm (SD = 0.026 mm), with values between 0.492 mm and 0.595 mm. Our proposed deep learning-based refinement method achieved an average SRE of 0.517 mm (SD = 0.025 mm), with extreme values of 0.482 mm and 0.545 mm. Statistical analysis indicated no significant differences in SRE across the three models (*p* = 0.072).

**Figure 8:**
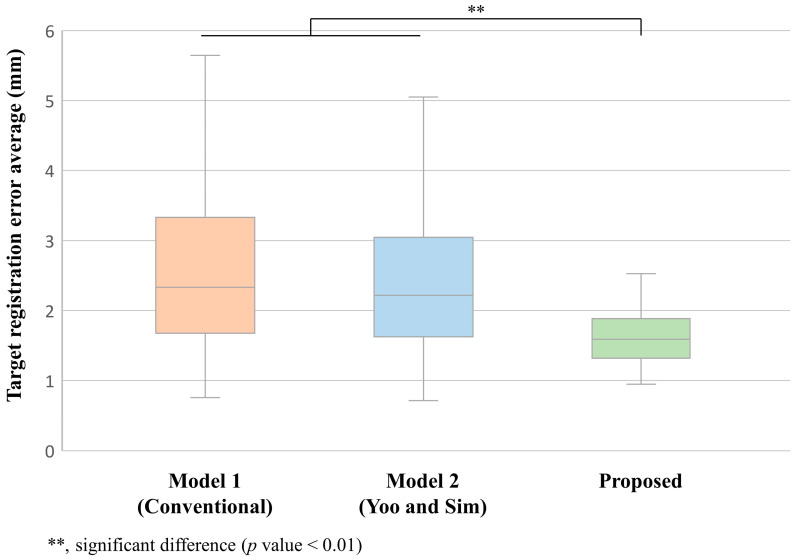
Box and whisker plots of TRE for three different registration pipelines: Conventional [15], Yoo and Sim [23], and the proposed method.

Box and whisker plots illustrate the distribution of TRE for three distinct registration pipelines (Figure 8). Model 1 displayed the highest median TRE (2.37 ± 1.14 mm, mean ± SD). Model 2 achieved a slightly improved median TRE of 2.29 ± 0.95 mm. The proposed deep learning-based refinement method yielded the lowest median TRE among all models (1.58 ± 0.52 mm), featuring the narrowest interquartile range. This method’s improvement was statistically significant compared to both the conventional approach and the technique by Yoo and Sim (*p* < 0.01). The whiskers on the plot, which depict the range of TRE values excluding outliers, were considerably shorter for our proposed method (range: 0.98–2.54 mm) than for Model 1 (range: 0.82–5.68 mm) and Model 2 (range: 0.78–5.06 mm).

**Figure 9:**
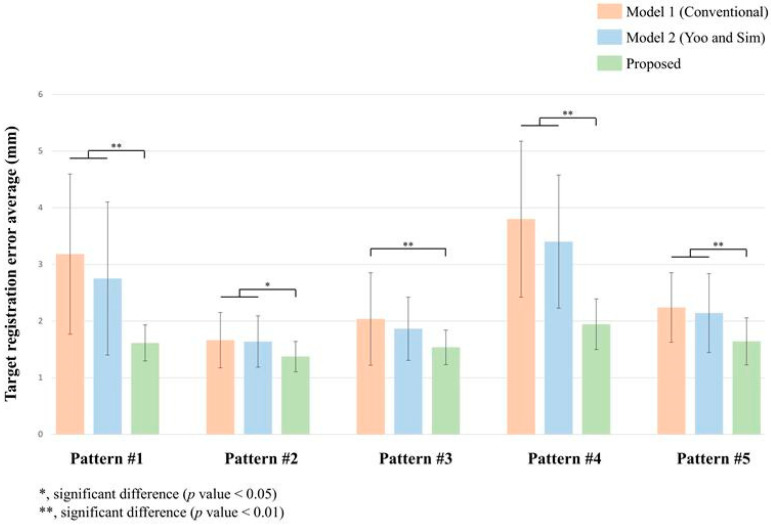
Bar graph of TRE distributions across five distinct patient surface point cloud patterns for three registration pipelines: Conventional [15], Yoo and Sim [23], and the proposed method.

Figure 9 illustrates the TRE distributions across five unique patient surface point cloud patterns. Patterns #1 (Forehead + nose), #4 (Forehead), and #5 (Above lip) exhibited highly significant differences with the proposed model (*p* < 0.01) compared to both Model 1 and Model 2. For Pattern #1, the proposed model recorded a mean TRE of 1.62 ± 0.32 mm, a 49.2% reduction relative to Model 1 (3.18 ± 1.41 mm) and a 41.3% reduction compared to Model 2 (2.75 ± 1.35 mm). Pattern #4 experienced a mean TRE of 1.95 ± 0.45 mm with the proposed model, which represents a 48.7% reduction from Model 1 (3.80 ± 1.38 mm) and a 42.8% reduction from Model 2 (3.40 ± 1.18 mm). In Pattern #5, the proposed model achieved a mean TRE of 1.64 ± 0.41 mm, showing a 26.8% reduction from Model 1 (2.24 ± 0.61 mm) and a 23.4% reduction from Model 2 (2.14 ± 0.70 mm). Pattern #2 (Entire face) revealed statistically significant differences (*p* < 0.05) between the proposed model and both Model 1 and Model 2. The proposed model achieved a mean TRE of 1.37 ± 0.27 mm, a 17.5% reduction from Model 1 (1.66 ± 0.49 mm) and a 16.5% reduction from Model 2 (1.64 ± 0.45 mm). For Pattern #3 (Face borderline), the proposed model exhibited a mean TRE of 1.54 ± 0.31 mm, significantly different (*p* < 0.01) from Model 1 (2.04 ± 0.82 mm) with a 24.5% reduction. A 17.5% reduction was noted compared to Model 2 (1.86 ± 0.56 mm), though it was not statistically significant. Across all patterns, the proposed model showed consistently lower TRE values with smaller standard deviations compared to Model 1 and Model 2.

Table 3 presents the TRE for three distinct target regions: front, middle, and back. In the front region, the proposed model demonstrated a mean TRE of 1.220 ± 0.177 mm, significantly lower (*p* < 0.01) than both Model 1 (1.865 ± 0.994 mm) and Model 2 (1.738 ± 0.848 mm), representing reductions of 34.6% and 29.8%, respectively. For the middle region, the proposed model achieved a mean TRE of 1.591 ± 0.207 mm, again showing a significant improvement (*p* < 0.01) over Model 1 (2.315 ± 0.929 mm) and Model 2 (2.216 ± 0.784 mm), with reductions of 31.3% and 28.2%, respectively. In the back region, where the increased distance from the registered surface was most pronounced, the proposed model exhibited a mean TRE of 1.986 ± 0.260 mm, significantly lower (*p* < 0.01) than both Model 1 (3.308 ± 1.403 mm) and Model 2 (3.239 ± 1.321 mm), representing substantial improvements of 40.0% and 38.7%, respectively. The proposed model consistently outperformed Models 1 and 2, demonstrating lower standard deviations across all regions. Most notably, it achieved a sub-2 mm accuracy in the back region, despite the increased depth challenge. These findings underscore the proposed model’s substantial improvement in registration accuracy across all target regions, with enhancements ranging from 28.2% to 40.0% relative to existing methods.

## 4. Discussion

The widely-adopted ICP algorithm and its derivatives, commonly used for surface registration, often suffer from convergence to local optima and a high dependence on precise initialization [10,46]. This problem becomes particularly acute in the presence of noise and outliers, which are common in surgical settings. The challenges in effectively registering the surgical site to anatomical landmarks, alongside human errors in landmark localization and the scarcity of reliable landmarks, particularly limit the accuracy of surgical navigation systems that rely on surface registration. Our study addresses these obstacles by introducing a novel registration protocol that significantly enhances the accuracy of conventional surface registration methods. By incorporating a deep learning-based refinement step between the initial coarse registration and the ICP registration, our approach improves accuracy without disrupting established surgical workflows or adding complex procedures. Our results demonstrate that this refined method markedly reduces registration errors compared to traditional approaches.

The consistent performance of our model adds significant value, especially considering the practical limitations encountered in real surgical scenarios. Several factors limit the ability to obtain comprehensive facial point clouds during surgery [47,48]:

- Presence of respiratory apparatus: Endotracheal tubes and other respiratory support devices can obstruct access to the lower face, limiting point cloud acquisition.
- Mobility of the mandible: Due to its range of motion and the fact that it is not rigidly attached to the skull, the mandible can introduce variability in the geometry of the lower face during point cloud acquisition.
- Patient breathing: Respiratory motion can result in subtle but significant changes in facial geometry, especially in the mid and lower face regions.

These constraints often necessitate reliance on more stable, bony areas such as the forehead, nose bridge, and zygomatic regions [10,28]. Given these limitations, the ability of our model to maintain strong performance even with restricted patterns (1, 4, and 5), as shown in Figure 9, offers a notable advantage for practical surgical applications. Particularly, Pattern #4 (Forehead) exhibited the most significant improvement, with the proposed model achieving a mean TRE reduction of 48.7% compared to Model 1 and 42.8% compared to Model 2. This underscores the model’s effectiveness in cases where only a small range of the face is used for registration. Pattern #5 (above lip) also showed a relatively moderate improvement, with a 26.8% reduction compared to Model 1 and a 23.4% reduction compared to Model 2. The key innovation is to provide a refined starting point for the ICP algorithm. During ICP fine registration, the algorithm tends to prioritize regions with denser point clouds [46]. Our refinement step mitigates this problem by aligning the patient and medical image point clouds in relatively stable positions before the ICP stage. This intermediate step prevents the ICP algorithm from overfitting to areas with denser point clouds in the early stages of fine registration. Such overfitting is particularly problematic when dealing with restricted facial patterns, where uneven point cloud distribution can lead to biased registration and convergence on incorrect local minima.

The lack of significant differences in SRE across the three models (Table 2) can be attributed to the nature of the ICP algorithm used in the fine registration step. It is important to recognize that these similar SRE values do not necessarily imply equivalent accuracy in the overall registration. The ICP algorithm inherently finds the nearest points between two point clouds, even if they are not true correspondences [20]. This characteristic of the ICP may result in artificially low SRE values that do not accurately represent true alignment accuracy, particularly for deeper structures. A key factor affecting registration accuracy is the lever-arm effect [49]. This phenomenon occurs when small surface rotation errors are amplified with increasing distance from the registration surface. The effect becomes evident when examining the TRE at various depths (Table 3). In the front region, our proposed model achieved a mean TRE of 1.220 ± 0.177 mm, which increased to 1.986 ± 0.260 mm in the posterior region, representing an increase of 0.766 mm. In contrast, the TRE for Model 1 increased from 1.865 ± 0.994 mm to 3.308 ± 1.403 mm (an increase of 1.443 mm), while Model 2 showed a similar trend, increasing from 1.738 ± 0.848 mm to 3.239 ± 1.321 mm (an increase of 1.501 mm). The increase in TRE from the front to the back regions clearly demonstrates the effect of error propagation. Notably, our method maintained accuracy under 2 mm in the back region, resulting in significant improvements of 40.0% and 38.7% compared to Model 1 and Model 2, respectively (*p* < 0.01). This difference in TRE for deeper structures highlights how minor surface misalignments can lead to substantial inaccuracies at depth.

The ICP algorithm, by searching the neighboring points that best match, can converge on local minima, especially when landmark picking errors in conventional methods or translational errors over 2 mm in the Yoo and Sim’s approach provide incorrect initial conditions for fine registration. This improvement in deep structure alignment, despite similar surface errors, underscores the importance of considering volumetric accuracy rather than just surface matching in image-guided surgical systems. Our deep learning-based refinement method addresses this issue by refining the alignment and providing a more accurate search space for finding correspondences during the fine registration step [46]. By constraining the search to a more relevant region, our method enables the ICP algorithm to explore a more appropriate range of transformations. This leads to a global optimum that better represents the true anatomical alignment at all depths, effectively mitigating the impact of the lever-arm effect and allowing for more accurate alignment throughout the entire volume.

The design of the deep-learning model architecture is critical in the proposed registration protocol for surgical navigation systems. Unlike other technological fields where source point clouds, such as those obtained from lidar or scanners, are plentiful, point clouds in the patient space in surgical navigation systems are sparse [50]. This sparsity presents unique challenges, necessitating a specialized deep-learning model designed for the specific requirements of surgical navigation point cloud learning. Recent advances in deep learning, particularly the development of transformer architectures, have shown great promise in handling point cloud data [51]. Transformers, with their attention mechanisms, excel at capturing and interacting with complex spatial relationships and have demonstrated an impressive performance in various point cloud registration tasks [52,53]. These models typically exploit local geometric structures encoded by graph neural networks or continuous convolution-based methods [54,55], allowing them to effectively process and learn from detailed spatial information. However, while transformers are powerful in scenarios with rich, dense point cloud data, they may not be optimal for the sparse point clouds typical in surgical navigation [56]. In our case, where only a few anatomical landmark inputs are available, a different approach is needed to address the unique challenges posed by the limited and sparse nature of the data. To address these specific challenges, we propose a novel model architecture that is tailored to the sparse nature of surgical navigation point clouds. The proposed model architecture diverges from deep-learning models for point clouds that rely on local geometric structures. Instead, it employs a global feature-based approach, which is more suitable for the sparse nature of point clouds in surgical navigation. By focusing on global features, the model can effectively capture and learn from the limited information available, ensuring accurate registration despite the scarcity of data points. To enhance the model’s ability to process these global features, we incorporate feature interaction modules that facilitate the exchange of information across different network layers. These modules work in a similar manner to attention mechanisms, allowing the model to capture multi-scale dependencies and enhance its representational power [57]. This approach allows for effective processing of the sparse point cloud data by facilitating the exchange of feature information, optimized for our specific use case with limited data points. To further boost the model’s performance, several architectural features have been incorporated. The iterative prediction structure, akin to the ICP algorithm, enables the model to progressively refine its predictions, incorporating feedback from previous cycles [58]. By continuously updating the transformation parameters, the model can converge toward an optimal solution, thus enhancing the accuracy of the registration process. Skip connections also facilitate the reuse of features from earlier stages, thereby enhancing the network’s representational power [59]. Additionally, the model architecture includes a dual-branch design for the separate processing of rotational and translational components [60]. This strategy allows the model to independently learn and predict rotations and translations, preserving the distinct spatial characteristics of each component and making robust predictions.

Our deep-learning protocol offers several key advantages over Yoo and Sim’s approach. By utilizing the coarse-registered state as a starting point, our method dramatically reduces the learning space that the deep-learning model needs to cover for inference. This contrast with Yoo and Sim’s approach, which attempts to learn the entire transformation from unaligned spaces. Additionally, we used anatomical landmarks and neighboring regions of localization error instead of patient point cloud patterns, which enhances the generalization of the trained model. This approach allows our model to focus on refining already roughly aligned landmarks, rather than learn the entire separated patient and medical image space from scratch. The result is improved inference capabilities and better generalization to surgical scenarios, particularly in cases with limited or restricted facial data. This improvement is evident in our model’s overall performance, as shown in Figure 8. Our deep-learning method, while requiring substantial computational resources for preoperative training, offers significant advantages over conventional registration techniques during surgery. Both our approach and the traditional method typically require 40 to 50 s for the initial coarse registration [12,61]. However, conventional methods heavily rely on the operator’s expertise and may require multiple attempts to achieve a satisfactory level of accuracy [10,28]. In contrast, our model can make inferences within 250 ms, refining the coarse registration almost in real time and achieving optimal accuracy in a single attempt. This deep learning-based refinement reduces the total duration of the preparation, contributing to overall surgical efficiency.

Our study demonstrates significant improvements in registration accuracy; however, some limitations warrant consideration. First, the protocol was validated only with phantom models rather than actual patients. Although these phantoms mimic soft tissue properties, they may not accurately replicate the complex deformations and physiological motions found in real patients, potentially affecting the model’s performance in clinical settings. To overcome this limitation, future research should focus on validating the proposed method using data from actual surgical procedures. Validation could begin with a retrospective analysis of existing surgical datasets and prospective clinical studies implementing the proposed method alongside current navigation systems. Second, our study employed a specific deep-learning model architecture in the proposed registration strategy. Since our primary aim was to establish a new surface registration protocol, we did not extensively explore various model architectures. However, investigating newer model architectures and advanced deep-learning techniques could potentially improve the accuracy and efficiency of the training process. Future studies should focus on comparing different deep-learning models and these emerging techniques to identify the most effective strategies for enhancing overall performance.

## 5. Conclusions

This study introduces a novel deep learning-based refinement for enhancing surface registration accuracy in surgical navigation systems. By integrating this approach between conventional coarse registration and ICP fine registration, we demonstrate significant improvements in registration precision while seamlessly incorporating the process into existing clinical workflows. Our findings show statistically significant reductions in TREs (average TRE: 1.58 ± 0.52 mm) compared to both conventional methods (2.37 ± 1.14 mm) and the recent deep-learning approach by Yoo and Sim [23] (2.29 ± 0.95 mm) (*p* < 0.01). This advancement has substantial implications for clinical trials and practice. The improved accuracy could potentially reduce surgical complications, enable more precise minimally invasive procedures, decrease procedure times, and enhance outcome consistency across various medical specialties. While the current focus on craniofacial applications has demonstrated the protocol’s effectiveness, the underlying principles are adaptable to other surgical domains, including spine, orthopedic, and pelvic surgeries. As surgical navigation technology continues to evolve, the integration of sophisticated computational techniques, such as the one presented here, may play an increasingly supportive role in enhancing existing processes without disrupting established procedures, ultimately contributing to improved surgical outcomes and patient care.

## Figures and Tables

**Table 1:** RMSE values for the predicted rotation angles and translation values across five different folds.

Fold Number	Rotation Angle RMSE (deg.)			Translation RMSE (mm)		
	Rx	Ry	Rz	Tx	Ty	Tz
1	0.989	0.726	1.089	0.334	0.695	0.753
2	0.976	0.837	1.114	0.566	0.683	0.619
3	1.067	0.850	1.043	0.520	0.774	0.715
4	1.105	0.812	1.118	0.365	0.695	0.715
5	1.051	0.810	1.105	0.610	0.641	0.732
Average	1.037	0.807	1.094	0.479	0.698	0.706
Standard Deviation (SD)	0.054	0.048	0.031	0.123	0.048	0.052

**Table 2:** Comparison of SRE after ICP fine registration.

	Model 1 (Conventional)				Model 2 (Yoo and Sim [23])				Proposed			
	Min	Max	Average	SD	Min	Max	Average	SD	Min	Max	Average	SD
SRE (mm)	0.482	0.596	0.534	0.025	0.492	0.595	0.544	0.026	0.482	0.545	0.517	0.025

**Table 3:** TRE by target region for three models.

Model Name	TRE by Target Region (mm)		
	Front	Middle	Back
Model 1 (Conventional)	1.865 ± 0.994	2.315 ± 0.929	3.308 ± 1.403
Model 2 (Yoo and Sim [23])	1.738 ± 0.848	2.216 ± 0.784	3.239 ± 1.321
Proposed	1.220 ± 0.177 ^a,b^	1.591 ± 0.207 ^a,b^	1.986 ± 0.260 ^a,b^

^a^ Significant difference (*p* < 0.01) between the proposed model and Model 1; ^b^ Significant difference (*p* < 0.01) between the proposed model and Model 2.

## Data Availability

The data presented in this study are available on request from the corresponding author.

## References

[1] Cleary K., Peters T.M. (2010). Image-guided interventions: Technology review and clinical applications. Annu. Rev. Biomed. Eng..

[2] Peters B.S., Armijo P.R., Krause C., Choudhury S.A., Oleynikov D. (2018). Review of emerging surgical robotic technology. Surg. Endosc..

[3] McCrory B., LaGrange C.A., Hallbeck M.S. (2014). Quality and safety of minimally invasive surgery: Past, present, and future. Biomed. Eng. Comput. Biol..

[4] Reddy K., Gharde P., Tayade H., Patil M., Reddy L.S., Surya D., Srivani Reddy L., Surya D. (2023). Advancements in robotic surgery: A comprehensive overview of current utilizations and upcoming frontiers. Cureus.

[5] Mezger U., Jendrewski C., Bartels M. (2013). Navigation in surgery. Langenbeck Arch. Surg..

[6] Azarmehr I., Stokbro K., Bell R.B., Thygesen T. (2017). Surgical navigation: A systematic review of indications treatments and outcomes in oral and maxillofacial surgery. J. Oral Maxillofac. Surg..

[7] Alam F., Rahman S.U., Ullah S., Gulati K. (2018). Medical image registration in image guided surgery: Issues, challenges and research opportunities. Biocybern. Biomed. Eng..

[8] Chang C.M., Jaw F.S., Lo W.C., Fang K.M., Cheng P.W. (2016). Three-dimensional analysis of the accuracy of optic and electromagnetic navigation systems using surface registration in live endoscopic sinus surgery. Rhinology.

[9] Mongen M.A., Willems P.W. (2019). Current accuracy of surface matching compared to adhesive markers in patient-to-image registration. Acta Neurochir..

[10] Eggers G., Mühling J., Marmulla R. (2006). Image-to-patient registration techniques in head surgery. Int. J. Oral Maxillofac. Surg..

[11] Khalifa F., Beache G.M., Gimel’farb G., Suri J.S., El-Baz A.S. (2011). State-of-the-art medical image registration methodologies: A survey. Multi Modality State-of-the-Art Medical Image Segmentation and Registration Methodologies.

[12] Fan Y., Yao X., Xu X. (2020). A robust automated surface-matching registration method for neuronavigation. Med. Phys..

[13] Fan Y., Jiang D., Wang M., Song Z. (2014). A new markerless patient-to-image registration method using a portable 3D scanner. Med. Phys..

[14] Li W., Fan J., Li S., Zheng Z., Tian Z., Ai D., Song H., Chen X., Yang J. (2023). An incremental registration method for endoscopic sinus and skull base surgery navigation: From phantom study to clinical trials. Med. Phys..

[15] Besl P.J., McKay N.D. (1992). A method for registration of 3-D shapes. IEEE Trans. Pattern Anal. Mach. Intell..

[16] Li P., Wang R., Wang Y., Tao W. (2020). Evaluation of the ICP algorithm in 3D point cloud registration. IEEE Access.

[17] Jiang L., Zhu J., Fei B., Liu J., Cheung Y.M., Ye X., Engelhardt K., Zhang J. (2015). A robust automated markerless registration framework for neurosurgery navigation. Int. J. Med. Robot. Comput. Assist. Surg..

[18] Qi C.R., Su H., Mo K., Guibas L.J. Pointnet: Deep learning on point sets for 3d classification and segmentation. Proceedings of the IEEE Conference on Computer Vision and Pattern Recognition.

[19] Wang Y., Sun Y., Liu Z., Sarma S.E., Bronstein M.M., Solomon J.M. (2019). Dynamic graph cnn for learning on point clouds. ACM Trans. Graph..

[20] Li Y., Bu R., Sun M., Wu W., Di X., Chen B. (2018). Pointcnn: Convolution on x-transformed points. Adv. Neural Inf. Process. Syst..

[21] Unberath M., Gao C., Hu Y., Judish M., Taylor R.H., Armand M., Grupp R. (2021). The impact of machine learning on 2d/3d registration for image-guided interventions: A systematic review and perspective. Front. Robot. AI.

[22] Ali M., Pena R.M.G., Ruiz G.O., Ali S. (2022). A comprehensive survey on recent deep learning-based methods applied to surgical data. arXiv.

[23] Yoo H., Sim T. (2022). A Deep Learning-Based Approach for Automated Coarse Registration (ACR) of Image-Guided Surgical Navigation. IEEE Access.

[24] Lorensen W.E., Cline H.E. Marching cubes: A high resolution 3D surface construction algorithm. Proceedings of the 14th Annual Conference on Computer Graphics and Interactive Techniques—SIGGRAPH ’87.

[25] Arun K.S., Huang T.S., Blostein S.D. (1987). Least-squares fitting of two 3-D point sets. IEEE Trans. Pattern Anal. Mach. Intell..

[26] Shamir R.R., Joskowicz L., Shoshan Y. (2011). Fiducial Optimization for Minimal Target Registration Error in Image-Guided Neurosurgery. IEEE Trans. Med. Imaging.

[27] Woerdeman P.A., Willems P.W., Noordmans H.J., Tulleken C.A., van der Sprenkel J.W. (2007). Application accuracy in frameless image-guided neurosurgery: A comparison study of three patient-to-image registration methods. J. Neurosurg..

[28] Taleb A., Guigou C., Leclerc S., Lalande A., Bozorg Grayeli A. (2023). Image-to-patient registration in computer-assisted surgery of head and neck: State-of-the-art, perspectives, and challenges. J. Clin. Med..

[29] Omara A., Wang M., Fan Y., Song Z. (2014). Anatomical landmarks for point-matching registration in image-guided neurosurgery. Int. J. Med. Robot. Comput. Assist. Surg. MRCAS.

[30] Wen A., Zhu Y., Xiao N., Gao Z., Zhang Y., Wang Y., Wang S., Zhao Y. (2023). Comparison Study of Extraction Accuracy of 3D Facial Anatomical Landmarks Based on Non-Rigid Registration of Face Template. Diagnostics.

[31] Fagertun J., Harder S., Rosengren A., Moeller C., Werge T., Paulsen R.R., Hansen T.F. (2014). 3D facial landmarks: Inter-operator variability of manual annotation. BMC Med. Imaging.

[32] Chabanas M., Luboz V., Payan Y. (2003). Patient specific finite element model of the face soft tissues for computer-assisted maxillofacial surgery. Med. Image Anal..

[33] Roccetti M., Delnevo G., Casini L., Cappiello G. (2019). Is bigger always better? A controversial journey to the center of machine learning design, with uses and misuses of big data for predicting water meter failures. J. Big Data.

[34] Xu B., Wang N., Chen T., Li M. (2015). Empirical evaluation of rectified activations in convolutional network. arXiv.

[35] Sarode V., Li X., Goforth H., Aoki Y., Srivatsan R.A., Lucey S., Choset H. (2019). Pcrnet: Point cloud registration network using pointnet encoding. arXiv.

[36] Wang Y., Solomon J.M. (2019). Prnet: Self-supervised learning for partial-to-partial registration. Advances in Neural Information Processing Systems.

[37] Fan H., Su H., Guibas L.J. A Point Set Generation Network for 3D Object Reconstruction from A Single Image. Proceedings of the IEEE Conference on Computer Vision and Pattern Recognition.

[38] Gojcic Z., Zhou C.F., Wegner J.D., Wieser A. The perfect match: 3D point cloud matching with smoothed densities. Proceedings of the 2019 IEEE/CVF Conference on Computer Vision and Pattern Recognition.

[39] Srinivasan K., Cherukuri A.K., Vincent D.R., Garg A., Chen B.Y. (2019). An efficient implementation of artificial neural networks with K-fold cross-validation for process optimization. J. Internet Technol..

[40] Chauhan T., Palivela H., Tiwari S. (2021). Optimization and fine-tuning of DenseNet model for classification of COVID-19 cases in medical imaging. Int. J. Inf. Manag. Data Insights.

[41] Guiotti A.M., Goiato M.C., Dos Santos D.M. (2010). Evaluation of the Shore A Hardness of Silicone for Facial Prosthesis as to the Effect of Storage Period and Chemical Disinfection. J. Craniofac. Surg..

[42] Herregodts S., Verhaeghe M., De Coninck B., Forward M., Verstraete M.A., Victor J., De Baets P. (2021). An improved method for assessing the technical accuracy of optical tracking systems for orthopaedic surgical navigation. Int. J. Med. Robot. Comput. Assist. Surg..

[43] Raabe A., Krishnan R., Wolff R., Hermann E., Zimmermann M., Seifert V. (2002). Laser surface scanning for patient registration in intracranial image-guided surgery. Neurosurgery.

[44] de Geer A.F., de Koning S.B., van Alphen M.J.A., Van der Mierden S., Zuur C.L., Van Leeuwen F.W.B., Loeve A.J., van Veen R.L., Karakullukcu M.B. (2022). Registration methods for surgical navigation of the mandible: A systematic review. Int. J. Oral Maxillofac. Surg..

[45] Lee S., Lee D.K. (2018). What is the proper way to apply the multiple comparison test?. Korean J. Anesthesiol..

[46] He Y., Liang B., Yang J., Li S., He J. (2017). An iterative closest points algorithm for registration of 3D laser scanner point clouds with geometric features. Sensors.

[47] Bobek S.L. (2014). Applications of navigation for orthognathic surgery. Oral Maxillofac. Surg. Clin. N. Am..

[48] Paydarfar J.A., Wu X., Halter R.J. (2019). Initial experience with image-guided surgical navigation in transoral surgery. Head Neck.

[49] Miga M.I., Sinha T.K., Cash D.M., Galloway R.L., Weil R.J. (2003). Cortical surface registration for image-guided neurosurgery using laser-range scanning. IEEE Trans. Med. Imaging.

[50] Wang M.N., Song Z.J. (2011). Properties of the target registration error for surface matching in neuronavigation. Comput. Aided Surg..

[51] Guo M.H., Cai J.X., Liu Z.N., Mu T.J., Martin R.R., Hu S.M. (2021). Pct: Point cloud transformer. Comput. Vis. Media.

[52] Qin Z., Yu H., Wang C., Guo Y., Peng Y., Xu K. Geometric transformer for fast and robust point cloud registration. Proceedings of the IEEE/CVF Conference on Computer Vision and Pattern Recognition.

[53] Yew Z.J., Lee G.H. Regtr: End-to-end point cloud correspondences with transformers. Proceedings of the IEEE/CVF Conference on Computer Vision and Pattern Recognition.

[54] Shi W., Rajkumar R. Point-gnn: Graph neural network for 3d object detection in a point cloud. Proceedings of the IEEE/CVF Conference on Computer Vision and Pattern Recognition.

[55] Wang S., Suo S., Ma W.C., Pokrovsky A., Urtasun R. Deep Parametric Continuous Convolutional Neural Networks. Proceedings of the IEEE Computer Society Conference on Computer Vision and Pattern Recognition.

[56] Liu Z., Yang X., Tang H., Yang S., Han S. FlatFormer: Flattened Window Attention for Efficient Point Cloud Transformer. Proceedings of the IEEE/CVF Conference on Computer Vision and Pattern Recognition.

[57] Wu B., Ma J., Chen G., An P. Feature interactive representation for point cloud registration. Proceedings of the IEEE/CVF International Conference on Computer Vision.

[58] Yew Z.J., Lee G.H. Rpm-net: Robust point matching using learned features. Proceedings of the IEEE/CVF Conference on Computer Vision and Pattern Recognition.

[59] He K., Zhang X., Ren S., Sun J. Deep residual learning for image recognition. Proceedings of the IEEE Conference on Computer Vision and Pattern Recognition.

[60] Xu H., Ye N., Liu S., Zeng B., Liu S. FINet: Dual branches feature interaction for partial-to-partial point cloud registration. Proceedings of the Thirty-Sixth AAAI Conference on Artificial Intelligence.

[61] Woodworth B.A., Davis G.W., Schlosser R.J. (2005). Comparison of laser versus surface-touch registration for image-guided sinus surgery. Am. J. Rhinol..

